# 7-Day Follow-Up Appointments Following Discharge From a Psychiatric Hospital

**DOI:** 10.1192/bjo.2024.339

**Published:** 2024-08-01

**Authors:** Isra Mizher, Ifeoma Amamilo

**Affiliations:** Royal Cornhill Hospital, Aberdeen, United Kingdom

## Abstract

**Aims:**

Following discharge from inpatient to community psychiatric services, the first 7 days is the most vulnerable and associated with an increased risk of suicide. According to the NICE Guideline 53, it is recommended that patients discharged from inpatient psychiatric services should be reviewed within 7 days by the relevant community services. Our aim was to determine how well we are adhering to this recommendation, appropriately documenting the appointment in the patients' discharge documents as well as the number of patients that attended the appointment.

**Methods:**

We collected data on an excel spreadsheet of patients discharged from Huntly ward (a General Adult Psychiatry ward) in the Royal Cornhill Hospital from 01/09/2022 and 14/10/2022 (a period of 6 weeks).

The data collected included name, CHI, date of admission and discharge, community mental health team, follow-up appointment offered, appropriate documentation on Core discharge document and whether the patient attended the appointment.

After the first audit cycle, we had a discussion with the junior doctors on the ward highlighting the importance of 7 day follow up and the need for arranging with the Community mental health team prior to the discharge, documenting a date, time and name of the clinician for the 7 day follow up in the Core discharge document. We also encouraged the use of reminders like using the doctors' diary book on the ward to document anticipated discharges and adequate hand over of patients to the community mental health team at the start of each week's Multidisciplinary Teams meeting.

We subsequently did a re-audit on patients discharged from Huntly ward between 04/04/2023 and 12/05/2023 (6 weeks). We compared the results from the first cycle and the second cycle to identify a change.

**Results:**

First Audit cycle.

Over the 6-week period, 27 patients were admitted into the Huntly ward and 23 patients were discharged.

48% (n = 11/23) of discharged patients were offered a follow up appointment.

91% (n = 10/11) had this appointment documented in the Core discharge document.

100% (n = 7/7) attended the 7 day follow up appointment.

Re-Audit.

Over the 6 week period, 16 patients were admitted and discharged from Huntly ward.

81% (n = 13/16) were offered a 7 day follow up appointment and this was documented in the Core discharge document.

100% (n = 13/13) of the patients attended their 7 day follow up appointment.

The result showed good improvement from 48% to 81%.

**Conclusion:**

Using reminders, properly liaising with the community mental health team, appropriately documenting a named clinician, date and time for the 7 day follow-up ensures that the patient attends.

The importance of offering support during the first week after discharge from psychiatric hospital should continue to be emphasized to prevent adverse outcome during this vulnerable period.

